# A Comparison of Urolithiasis in the Presence and Absence of Microscopic Hematuria in the Emergency Department

**DOI:** 10.5811/westjem.2017.4.33018

**Published:** 2017-05-15

**Authors:** Jason M. Mefford, Robert M. Tungate, Leila Amini, Dongjin Suh, Craig L. Anderson, Scott E. Rudkin, Megan Boysen-Osborn

**Affiliations:** * University of California, Davis, Department of Emergency Medicine, Sacramento, California; † University of Southern California, Keck School of Medicine, Los Angeles, California; ‡ University of California, Irvine School of Medicine, Department of Emergency Medicine, Irvine, California; § Virginia Commonwealth University School of Medicine, Richmond, Virginia

## Abstract

**Introduction:**

Urolithiasis is a common medical condition that accounts for a large number of emergency department (ED) visits each year and contributes significantly to annual healthcare costs. Urinalysis is an important screening test for patients presenting with symptoms suspicious for urolithiasis. At present there is a paucity of medical literature examining the characteristics of ureteral stones in patients who have microscopic hematuria on urinalysis versus those who do not. The purpose of this study was to examine mean ureteral stone size and its relationship to the incidence of clinically significant hydronephrosis in patients with and without microscopic hematuria.

**Methods:**

This is a retrospective chart review of patient visits to a single, tertiary academic medical center ED between July 1, 2008, and August 1, 2013, of patients who underwent non-contrast computed tomography of the abdomen and pelvis and urinalysis. For patient visits meeting inclusion criteria, we compared mean stone size and the rate of moderate-to-severe hydronephrosis found on imaging in patients with and without microscopic hematuria on urinalysis.

**Results:**

Out of a total of 2,370 patient visits 393 (16.6%) met inclusion criteria. Of those, 321 (82%) had microscopic hematuria present on urinalysis. Patient visits without microscopic hematuria had a higher rate of moderate-to-severe hydronephrosis (42%), when compared to patients with microscopic hematuria present (25%, p=.005). Mean ureteral stone size among patient visits without microscopic hematuria was 5.7 mm; it was 4.7 mm for those patients with microscopic hematuria (p=.09). For ureteral stones 5 mm or larger, the incidence of moderate-to-severe hydronephrosis was 49%, whereas for ureteral calculi less than 5 mm in size, the incidence of moderate-to-severe hydronephrosis was 14% (p < 0.0001).

**Conclusion:**

Patients visiting the ED with single-stone ureterolithiasis without microscopic hematuria on urinalysis could be at increased risk of having moderate-to-severe hydronephrosis compared to similar patients presenting with microscopic hematuria on urinalysis. Although the presence of hematuria on urinalysis is a moderately sensitive screening test for urolithiasis, these results suggest patients without hematuria tend to have more clinically significant ureteral calculi, making their detection more important. Clinicians should maintain a high index of suspicion for urolithiasis, even in the absence of hematuria, since ureteral stones in these patients were found to be associated with a higher incidence of obstructive uropathy.

## INTRODUCTION

Urolithiasis is a very common medical condition that affects 5–15% of the worldwide population^1^ and results in over 1.2 million emergency department (ED) visits in the United States each year.^2^ An important goal in the evaluation of urolithiasis is the detection of concomitant ureteral obstruction, which can result in irreversible renal damage and be associated with life-threatening infection. The evaluation of urolithiasis is largely influenced by the results of a urinalysis (UA). While the presence of microscopic hematuria favors a diagnosis of urolithiasis in a patient presenting with symptoms suggestive of ureteral colic, it is estimated that 10–20% of patients with urolithiasis can present without microscopic hematuria on UA.^3^ To the best of our knowledge, there are no large studies examining whether the presence or absence of microscopic hematuria has any influence on the likelihood of a patient having concomitant clinically significant hydronephrosis. In this study, we sought to compare the rates and severity of hydronephrosis in patients with non-contrast computed tomography-(CT) diagnosed urolithiasis in the presence and absence of microscopic hematuria on urinalysis.

## METHODS

We conducted a retrospective chart review of patient visits to a single, tertiary academic medical center ED between July 1, 2008, and August 1, 2013. We complied with optimal methods for retrospective chart reviews.^4^ All patient visits in adults aged 18 years or older that included a non-contrast CT of the abdomen and pelvis (CT abd/pelvis) and microscopic UA within this time frame met inclusion criteria. We excluded patient visits with any of the following: absence of ureteral calculi on non-contrast CT abd/pelvis radiology report; no UA data or missing UA data; missing non-contrast CT abd/pelvis radiology report; presence of more than one ureteral calculus; presence of a ureteral stent or nephrostomy tube; or presence of any intraabdominal or pelvic mass resulting in ureteral obstruction. We defined a ureteral stone as a calculus residing anywhere from the ureteropelvic junction to the ureterovesicular junction. All CTs were performed using either a Siemens Somatome Sensation 64-slice scanner, or a Philips Brilliance 128- or 256-slice scanner. A radiology faculty member at the University of California, Irvine Medical Center interpreted all CTs during the study period.

Two blinded, trained data abstractors (RT and DS) recorded the following on a data abstraction form: number of red blood cells (RBCs) per high power field (hpf) on UA; size and location of the ureteral stone; and the presence and severity of hydronephrosis (none, mild, moderate, or severe per attending radiology final report). We held periodic meetings to resolve any discrepancies and/or questions regarding the extraction of data from these reports. If the presence of hydronephrosis was not documented (n=39 charts) on the radiology report, we assumed that there was none. If hydronephrosis was documented, but without a clarifying severity (n=16 charts), we assumed that the patient fell into at least the moderate group. If the hydronephrosis was described as “mild to moderate” (n=19), we included these patients in the “moderate” hydronephrosis group. Two separate, non-blinded reviewers (MBO and JM) audited all included charts for accuracy.

**Population Health Research Capsule**

What do we already know about this issue?

Urolithiasis is a very common emergency department (ED) diagnosis accounting for a large number of ED visits in the U.S. each year.

What was the research question?

How does the rate and severity of hydronephrosis in CT-diagnosed urolithiasis compare between ED patients with and without microscopic hematuria on urinalysis?

What was the major finding of the study?

ED patients with CT-diagnosed urolithiasis and an absence of microscopic hematuria on urinalysis had a significantly higher rate of moderate-to-severe hydronephrosis compared to patients with microscopic hematuria on urinalysis.

How does this improve population health?

Emergency physicians should maintain a high index of suspicion for clinically significant urolithiasis despite an absence of microscopic hematuria on urinalysis in patients presenting to the ED with symptoms of renal colic.

We divided patient visits into two groups based on the presence or absence of microscopic hematuria on UA, as defined by guidelines established by the American Urological Association.^5^,^6^ We considered a UA with equal to or greater than four RBC per hpf to have microscopic hematuria present, and fewer than four RBC per hpf to be absent microscopic hematuria.

We calculated the average ureteral stone size, the incidence of moderate-to-severe hydronephrosis, and the incidence of any level of hydronephrosis (“minimal” or greater) for each of our patient groups (those with and those without hematuria). We performed all calculations using Microsoft Excel or Vassar Stats.^7^

## RESULTS

Out of a total of 2,370 patient visits that we reviewed, 393 met inclusion criteria. The median age of our patient population was 43 years (range: 18–91, interquartile range [IQR] [32–54]) and 69% were male. Among these, 321 (82%) had concomitant microscopic hematuria and 72 (18%) did not have microscopic hematuria on UA.

A higher proportion of patient visits without hematuria had moderate-to-severe hydronephrosis (n = 30, 42%) when compared to those with hematuria (n = 81, 25%) (p = .005 via chi-squared, negative likelihood ratio = 1.8) Stated another way, and acknowledging the limitations of the narrow patient population studied, the sensitivity of hematuria on urinalysis for detecting a ureteral calculus was 73% (95% confidence interval [CI] [64%–81%]) in the group of patients with moderate-to-severe hydronephrosis and 85% (95% CI [80%–89%]) in patients with mild or no hydronephrosis (p = .005). See Figure for a summary of results.

There was no difference in the proportion of patient visits with any amount of hydronephrosis (minimal, mild, moderate, or severe) with microscopic hematuria (n = 288, 90%) versus without microscopic hematuria (n = 65, 90%) (p = 0.92). The average ureteral stone size among all patients was 4.9 mm. The average size of ureteral stones for patient visits with microscopic hematuria was 4.7 mm (95% CI [4.4–5.0**;** range 1–20]) and 5.7 mm (95% CI [4.6–6.7; range 1–25]) in patient visits without microscopic hematuria, (p = 0.09 via two tailed t-test). For those patients with no, minimal, or mild hydronephrosis, the average stone size was 4.1 mm (CI [3.8–4.4, range: 1–20]); for those patients with moderate-to-severe hydronephrosis, the average stone size was 6.9 mm (CI [6.1–7.7, range: 1–25]) (p < 0.0001 via two tailed t-test). For ureteral calculi equal or greater than 5 mm in size, the incidence of moderate-to-severe hydronephrosis was 49%, whereas for ureteral calculi less than 5 mm in size, the incidence of moderate or more severe hydronephrosis was 14% (p < 0.0001).

## DISCUSSION

Urolithiasis is a very common diagnosis in the ED accounting for 5%–8% of ED visits and adding up to $5 billion in healthcare costs annually in the United States.^8^ While most ureteral stones will pass without consequence, the challenge for emergency physicians (EP) is to identify those patients who are at higher risk for complications, such as obstructive uropathy. Microscopic hematuria on UA is a good screening test in the workup of suspected ureteral colic, but its sensitivity ranges between 69% and 84%,^3^,^9^ similar to the rate found in our study of 82%.

Our retrospective chart review demonstrated that microscopic hematuria was less sensitive in detecting urolithiasis in patients with more severe disease (obstructive uropathy). It is unclear why a greater degree of obstructive uropathy would correlate with a lower incidence of microscopic hematuria. One hypothesis is that larger ureteral stones may obstruct bleeding resulting in the absence of hematuria on UA; however, no studies have proven this. Additional factors that may influence or confound the presence or absence of microscopic hematuria on UA in patients with suspected urolithiasis include dehydration, females on their menstrual period, stone position,^10^ and the time interval between pain onset and urine collection.^12^ A recent study by Sahin et al. examined the value of several parameters for predicting successful medical expulsion therapy in urolithiasis and found stone size, localization, degree of hydronephrosis, proximal ureteral diameter and ureteral wall thickness to be highly predictive, and patient age, BMI and stone density not predictive.^13^ Regardless, EPs may want to exercise caution in the management of patients with suspected ureteral colic without microscopic hematuria on UA, as our findings suggest these patients are at increased risk of more severe hydronephrosis.

The presence or absence of microscopic hematuria on UA is a point of interest, as its absence may prompt EPs to order more diagnostic CTs to narrow the differential diagnoses. At present, non-contrast helical CTs are the criterion reference of urinary stone diagnosis, with a measured sensitivity of 97–100%, specificity of 94–96%, and negative predicative value of 97%.^14^–^16^ However, non-contrast CT urography can underestimate ureteral stone size by up to 12%.^17^ CTs are also expensive, increase ED lengths of stay, and expose patients to ionization radiation.^20^–^23^ The expense can be further inflated by the workup of incidental and unrelated findings found on CT.^11^,^18^,^19^

We did not find a statistically significant difference between ureteral stone size in patients with and without microscopic hematuria. Our sample size may have been too small to detect one, although previous studies have also failed to demonstrate a significant correlation between stone size and the presence of hematuria.^10^ We did find, however, a significant difference in the mean size of ureteral stones resulting in minimal-to-mild hydronephrosis (4.1mm) versus those resulting in moderate-to-severe hydronephrosis (6.9 mm, p < 0.0001). Furthermore, stones that were 5mm or larger were associated with a higher incidence of moderate-to-severe hydronephrosis (49%) than those stones that were smaller than 5mm (14%, p < 0.0001). These findings suggest that the severity of an obstructive complication may increase significantly with ureteral stones around 5 mm in diameter or larger. This knowledge carries important clinical implications as it might aid EPs in better estimating a patient’s likelihood of an obstructive complication and consequently whether or not urological consultation is warranted.

## LIMITATIONS

Limitations of our study include its retrospective nature, though it strictly adheres to methods designed to minimize bias in emergency medicine retrospective chart reviews as outlined by Gilbert, Lowenstein, et al.^4^ We also examined a narrow patient population of ED visits with CT-proven urolithiasis only. This was intended to ensure all patients included in the study had direct visual evidence a ureteral calculus, however at the expense of excluding all patients clinically diagnosed with ureterolithiasis (i.e., no CT obtained). EPs are more likely to obtain a CT for patients with renal colic symptoms and no hematuria on UA given greater diagnostic uncertainty, and thus a selection bias for patients without hematuria on UA may have skewed our patient sample. Furthermore, the growing use of point-of-care ultrasound as an alternative imaging modality for diagnosing hydronephrosis at the bedside with reported sensitivities of 85–94% and specificity of 100% contributed additional confounding as these patients too were excluded if no CT was obtained.^24^–^26^ Some patient visits over the data collection time period may have been repeat visits by the same patient. Several patient visits that otherwise would have met inclusion criteria were excluded based on the absence of either UA data or a CT urography report. Some CT urography reports neglected to qualify the degree of hydronephrosis, and others varied in the verbiage used to describe the degree of hydronephrosis. Additionally several patient visits were excluded on the basis of having more than one ureteral stone seen on CT urography given that if hematuria were present on UA it could not be attributed to any one stone.

## CONCLUSION

Patient visits to the ED with a single ureteral stone on non-contrast CT abd/pelvis and no microscopic hematuria on UA are more likely to have moderate-to-severe concomitant hydronephrosis than patient visits with microscopic hematuria on UA. Future study should focus on patient-centered outcomes among those found to have clinically significant hydronephrosis without microscopic hematuria on urinalysis in order to better guide the workup and prognostication of this patient group. Additionally, further scientific investigation into the pathophysiological mechanisms responsible for hematuria in urolithiasis would greatly benefit physician interpretation of microscopic hematuria in this patient population.

## Figures and Tables

**Figure:**
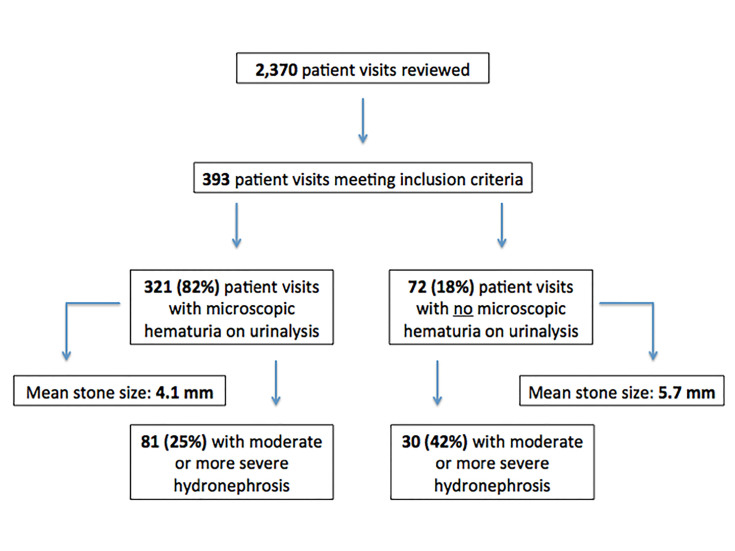
Summary of results including total number of patient visits reviewed, number of patient visits meeting inclusion criteria, percentage of included patient visits with and without microscopic hematuria on urinalysis, mean stone size and percentage of moderate-to-severe hydronephrosis among included patient visits with and without microscopic hematuria on urinalysis.

## References

[1] Moe OW (2006). Kidney stones: pathophysiology and medical management. Lancet.

[2] Eaton SH, Cashy J, Pearl JA (2013). Admission rates and costs associated with emergency presentation of urolithiasis: analysis of the Nationwide Emergency Department Sample 2006–2009. J Endourol.

[3] Luchs JS, Katz DS, Lane MJ (2002). Utility of hematuria testing in patients with suspected renal colic: correlation with unenhanced helical CT results. Urology.

[4] Gilbert EH, Lowenstein SR, Koziol-McLain J (1996). Chart reviews in emergency medicine research: Where are the methods?. Ann Emerg Med.

[5] (2007). Campbell-Walsh Urology.

[6] Grossfeld GD, Wolf JS, Litwin MS (2001). Asymptomatic microscopic hematuria in adults: Summary of the AUA Best Practice Policy recommendations. Am Fam Physician.

[7] VassarStats (1998–2016). http://vassarstats.net.

[8] Ghani KR, Roghmann F, Sammon JD (2014). Emergency department visits in the United States for upper urinary tract stones: trends in hospitalization and charges. J Urol.

[9] Eray O, Çubuk MS, Oktay C (2003). The efficacy of urinalysis, plain films, and spiral CT in ED patients with suspected renal colic. Am J Emerg Med.

[10] Inci MF, Ozkan F, Bozkurt S (2013). Correlation of volume, position of stone, and hydronephrosis with microhematuria in patients with solitary urolithiasis. Med Sci Monit.

[11] Li J, Kennedy D, Levine M (2001). Absent hematuria and expensive computerized tomography: case characteristics of emergency urolithiasis. J Urol.

[12] Kobayashi T, Nishizawa K, Mitsumori K (2003). Impact of date of onset on the absence of hematuria in patients with acute renal colic. J Urol.

[13] Sahin C, Eryildirim B, Kafkasli A (2015). Predictive parameters for medical expulsive therapy in ureteral stones: a critical evaluation. Urolithiasis.

[14] Jindal G, Ramchandani P (2007). Acute flank pain secondary to urolithiasis: Radiologic evaluation and alternate diagnoses. Radiol Clin North Am.

[15] Pfister SA, Deckart A, Laschke S (2007). Unenhanced helical computed tomography vs intravenous urography in patients with acute flank pain: accuracy and economic impact in a randomized prospective trial. Eur Radiol.

[16] Pearle MS, Calhoun EA, Curhan GC (2005). Urologic diseases in America project: urolithiasis. J Urol.

[17] Dundee P, Bouchier-Hayes D, Haxhimolla H (2006). Renal tract calculi: comparison of stone size on plain radiography and noncontrast spiral CT scan. J Endourol.

[18] Lumbreras B, Donat L, Hernández-Aguado I (2010). Incidental findings in imaging diagnostic tests: a systematic review. Br J Radiol.

[19] Thompson RJ, Wojcik SM, Grant WD (2011). Incidental findings on CT scans in the emergency department. Emerg Med Int.

[20] Quirke M, Divilly F, O’Kelly P (2011). Imaging patients with renal colic: a comparative analysis of the impact of non-contrast helical computed tomography versus intravenous pyelography on the speed of patient processing in the Emergency Department. Emerg Med J.

[21] Mathews JD, Forsythe AV, Brady Z (2013). Cancer risk in 680,000 people exposed to computed tomography scans in childhood or adolescence: data linkage study of 11 million Australians. BMJ.

[22] Pearce MS, Salotti JA, Little MP Radiation exposure from CT scans in childhood and subsequent risk of leukaemia and brain tumours: a retrospective cohort study. Lancet.

[23] Council NR (1998). Health Effects of Exposure to Low Levels of Ionizing Radiations: Time for Reassessment?.

[24] Smith-Bindman R, Aubin C, Bailitz J (2014). Ultrasonography versus computed tomography for suspected nephrolithiasis. N Engl J Med.

[25] Riddell J, Case A, Wopat R (2014). Sensitivity of emergency bedside ultrasound to detect hydronephrosis in patients with computed tomography-proven stones. West J Emerg Med.

[26] Kevin M, Ban JSE, Marx John A, Walls Ron M (2014). Selected Urologic Problems. Rosen’s Emergency Medicine: Concepts and Clinical Practice.

